# Outcome differences between PD-1/PD-L1 inhibitors-based monotherapy and combination treatments in NSCLC with brain metastases

**DOI:** 10.1186/s40164-023-00412-3

**Published:** 2023-06-23

**Authors:** Haowei Wang, Fangfang Liu, Xiaoxia Chen, Chao Zhao, Xuefei Li, Caicun Zhou, Jie Hu, Qian Chu, Tao Jiang

**Affiliations:** 1 Department of Medical Oncology, Shanghai Pulmonary Hospital, Tongji University School of Medicine, Zhengmin Road 507, Shanghai, 200433 China; 2 Department of Oncology, Tongji Hospital of Tongji Medical College, Huazhong University of Science and Technology, No. 288, Xintian Road, Wuhan, 430030 China; 3 Department of Lung Cancer and Immunology, Shanghai Pulmonary Hospital, Shanghai, China; 4 Department of Pulmonary Medicine, Zhongshan Hospital, Fudan University, No. 180, Fenglin Road, Shanghai, 200032 China

**Keywords:** Lung cancer, PD-1, Brain metastases, Tumor immune microenvironment

## Abstract

**Introduction:**

Without the clear immunophenotyping of brain metastases (BrMs), the optimal treatment strategy based on PD-1/PD-L1 inhibitor for patients with non-small-cell lung cancer (NSCLC) and BrMs remains unknown.

**Methods:**

308 patients with NSCLC received PD-1/PD-L1 inhibitor-based monotherapy or combination therapy were retrospectively identified. Kaplan-Meier curves with log-rank tests were used to determine the treatment outcomes differences. Transcriptomic analysis of paired primary lung lesions and BrMs were performed to dissect the specific tumor immune microenvironment (TIME) of BrMs.

**Results:**

The presence of BrMs was associated with significantly inferior PFS (2.5 vs. 3.7 months; *P* = 0.0053) and OS (8.3 vs. 15.4 months; *P* = 0.0122) in monotherapy group, while it was only associated with poorer PFS (4.6 vs. 7.0 months; *P* = 0.0009) but similar OS (22.8 vs. 21.0 months; *P* = 0.9808) in combination treatment group. Of patients with BrMs, PD-1/PD-L1 inhibitor plus antiangiogenic therapy was associated with longest PFS (7.7 vs. 3.2 vs. 2.5 months; *P* = 0.0251) and OS (29.2 vs. 15.8 vs. 8.3 months; *P* = 0.0001) when compared with PD-1/PD-L1 inhibitor plus chemotherapy or anti-PD-1/PD-L1 monotherapy. Multivariate analyses suggested that combination treatment was independently correlated with significantly longer PFS (*P* = 0.028) and OS (*P* < 0.001) in patients with BrMs. Transcriptomic analysis showed a suppressive TIME in BrMs with decreased CD4^+^ T cells and M1 macrophages but increased M2 macrophages infiltration.

**Conclusion:**

NSCLC with BrMs obtained barely satisfactory overall benefit from anti-PD-1/PD-L1 monotherapy, partly due to its immunosuppressive TIME. PD-1/PD-L1 inhibitor-based combination treatment, especially anti-PD-1/PD-L1 plus anti-angiogenic treatment, could significantly improve the clinical outcomes of patients with NSCLC and BrMs.

**Supplementary Information:**

The online version contains supplementary material available at 10.1186/s40164-023-00412-3.

## Introduction

Brain metastasis (BrM), one of the most common distant metastases in non-small-cell lung cancer (NSCLC), still remains an awkward disease with unsatisfactory overall prognosis [1–5]. BrMs occur in approximately 40% of patients with NSCLC during his/her lifetime [6, 7]. Radiotherapy remains to be the mainstay of local treatment in patients with NSCLC and BrMs. Recently, several systemic treatments, especially molecularly targeted therapy, have shown promising activity on BrMs with driver genes alterations due to the satisfactory central nervous system penetration [4, 5, 8]. However, most patients with BrMs but without driver gene alterations have very limited therapeutic options together with dismal long-term survival.

Immune checkpoint inhibitors (ICIs) including programmed cell death 1 (PD-1) and its ligand (PD-L1) inhibitors have significantly improved the prognosis and shifted the treatment paradigm in advanced NSCLC. However, patients with BrMs were often excluded from clinical trials, or only high-selected patients could be included [3, 4, 9]. Although several recent publications reported that anti-PD-1/PD-L1 monotherapy showed good activity for controlling BrMs, the efficacy was barely satisfactory [10]. Even some studies indicated that the presence of BrMs was correlated with inferior survival in patients with NSCLC received anti-PD-1/PD-L1 monotherapy [11, 12], suggesting an unmet treatment need for these populations. More recently, several clinical trials demonstrated that PD-1/PD-L1 inhibitors plus chemotherapy and/or antiangiogenic therapy could significantly prolong progression-free and overall survival (PFS and OS) in patients with advanced or metastatic NSCLC [13–15]. Subgroup analyses in those with BrMs suggested that anti-PD-1/PD-L1 based combination therapy could result in prolonged OS than chemotherapy [16]. However, these trials only included high-selected patients with BrMs (for example, untreated or asymptomatic BrMs) and their control group is often traditional chemotherapy, which was not well consistent with currently clinical practice. Thus, whether anti-PD-1/PD-L1 based combination treatments could show better efficacy than anti-PD-1/PD-L1 monotherapy in NSCLC patients with less-selected BrMs remains unknown.

Here, we performed this multicenter retrospective study to investigate the impact of BrMs on the efficacy of ICI based treatments in NSCLC, and the outcome differences between PD-1/PD-L1 inhibitor-based monotherapy and combination therapies in patients with NSCLC and BrMs. To dissect the specific tumor immune microenvironment (TIME) of BrMs and investigate the potential explanations for different treatment outcomes of anti-PD-1/PD-L1 based therapies, we conducted a transcriptomic analysis on paired samples from primary lung cancers and BrMs.

## Materials and methods

### Patients’ inclusion

We retrospectively reviewed the patients diagnosed with advanced or metastatic NSCLC who received PD-1/PD-L1 inhibitors-based treatments from February 1, 2017 to September 1, 2020 in three medical centers. The major inclusion criteria were (i) histological or pathological confirmation of metastatic or advanced NSCLC, (ii) radiological confirmation of BrM (at least one of brain lesions was measurable) including enhanced computed tomography (CT) and/or cranial magnetic resonance imaging (MRI), (iii) evaluable for treatment response assessment. The main exclusion criteria included (i) leptomeningeal metastases, (ii) previous cranial surgery, (iii) patients with severe CNS symptoms including uncontrollable intracranial hypertension or intracranial hemorrhage. Other distant metastases were detected by using thoracic and abdominal CT/MRI, whole body positron emission tomography (PET) or PET/CT, abdominal ultrasound or bone scan. All included patients received anti-PD-1/PD-L1 antibodies as monotherapy or plus chemotherapy or anti-angiogenesis or both, regardless of treatment lines. The dose of each type of anti-PD-1/PD-L1 antibodies and other antitumor drugs was used according to the recommended dose from drug instructions or phase II/III trials. The study protocol was approved by the ethics committee and institutional review board of each center. Patients who met the above-mentioned criteria were included from three centers with analogous standard therapeutic procedure for patients with NSCLC and BrMs.

### Data collection

We collected the data of eligible patients from electronic medical records by using the same requirements for clinical data on patient’s follow-up under treatment, including response to different treatments and clinical outcomes. The baseline parameters including age, sex, smoking history, Eastern Cooperative Oncology Group performance status (ECOG PS), lung cancer histology (WHO classification), driver gene alteration status, PD-L1 expression level, sites of extracranial metastasis, corticosteroid usage, symptoms at start of ICI treatment, therapeutic regimens, treatment lines and types of ICIs were collected. Age, ECOG PS and smoking status were recorded at initial diagnosis. A never smoker was defined as a person who had smoked < 100 cigarettes during his/her lifetime. Common driver genes alterations including *EGFR*, *HER2*, *KRAS*, *BRAF*, *ALK*, *ROS1* and *RET* were determined by amplification refractory mutation system or multiplex real-time polymerase chain reaction as described in our previous publications [6, 17, 18]. PD-L1 expression, as measured by the DAKO 22C3 assay using immunohistochemical staining, is defined as the percentage of viable tumor cells showing partial or complete membrane staining at any intensity (positive was defined as ≥ 1%). Test methods were conducted according to the manufacturer’s recommendations and our previous study [19]. Whether chemotherapy or anti-angiogenic therapy or both was added to anti-PD-1/PD-L1 antibody were selected according to clinical treatment guidelines or by the investigators’ or patients’ discretion. Tumor response was assessed one month after the initiation of therapy and then every two months based on the Response Evaluation Criteria in Solid Tumors (RECIST) version 1.1. Treatment response assessment included complete response (CR), partial response (PR), stable disease (SD) or progressive disease (PD). Last follow-up was January 1, 2022.

### Transcriptomic analysis

The raw RNA sequencing data (770 immune-related genes, NanoString nCounter PanCancer Immune Profiling Panel) of 22 samples from eleven paired primary lung cancers and brain metastasis was downloaded from a previous study [20]. The immune cell composition was calculated by the CIBERSORT algorithm [21]. The differential gene expression analysis were performed by using the DESseq2 package in R software (version 3.6.3), with adjusted *P* < 0.05 and |log_2_(Fold Change)| > 0.5. Output data were normalized using a negative binomial distribution statistical method. For each gene, the expression score was calculated as the expression levels [log2 (TPM + 1)] of this gene.

### Statistical analysis

Clinicopathologic characteristics were summarized by number and percentages. Chi-square test, or Fisher’s exact test when needed was used to compare the categorical variables. The continuous variables were analyzed by ANOVA and/or Tukey’s multiple comparison tests. The difference of clinicopathologic features between treatment groups was compared with the χ^2^ test. PFS was defined as the time from the date of initiation of ICIs based treatment to the date of systemic progression (including intracranial progression) or death and was censored at the date of last tumor assessment (when carried out). OS was calculated from the date of ICIs based treatment start to the date of death of any cause or last follow-up. The outcome differences were determined by using the Kaplan-Meier curves with two-sided log-rank tests and Cox proportional hazards model with calculated hazard ratios (HRs) and 95% confidence intervals (CIs). All statistical analyses were conducted by using the SPSS statistical software, version 22.0 (SPSS Inc., Chicago, IL, USA). Two-sided *P* values were considered significant at *P* < 0.05.

## Results

### Baseline features of all included patients

**Fig. 1:**
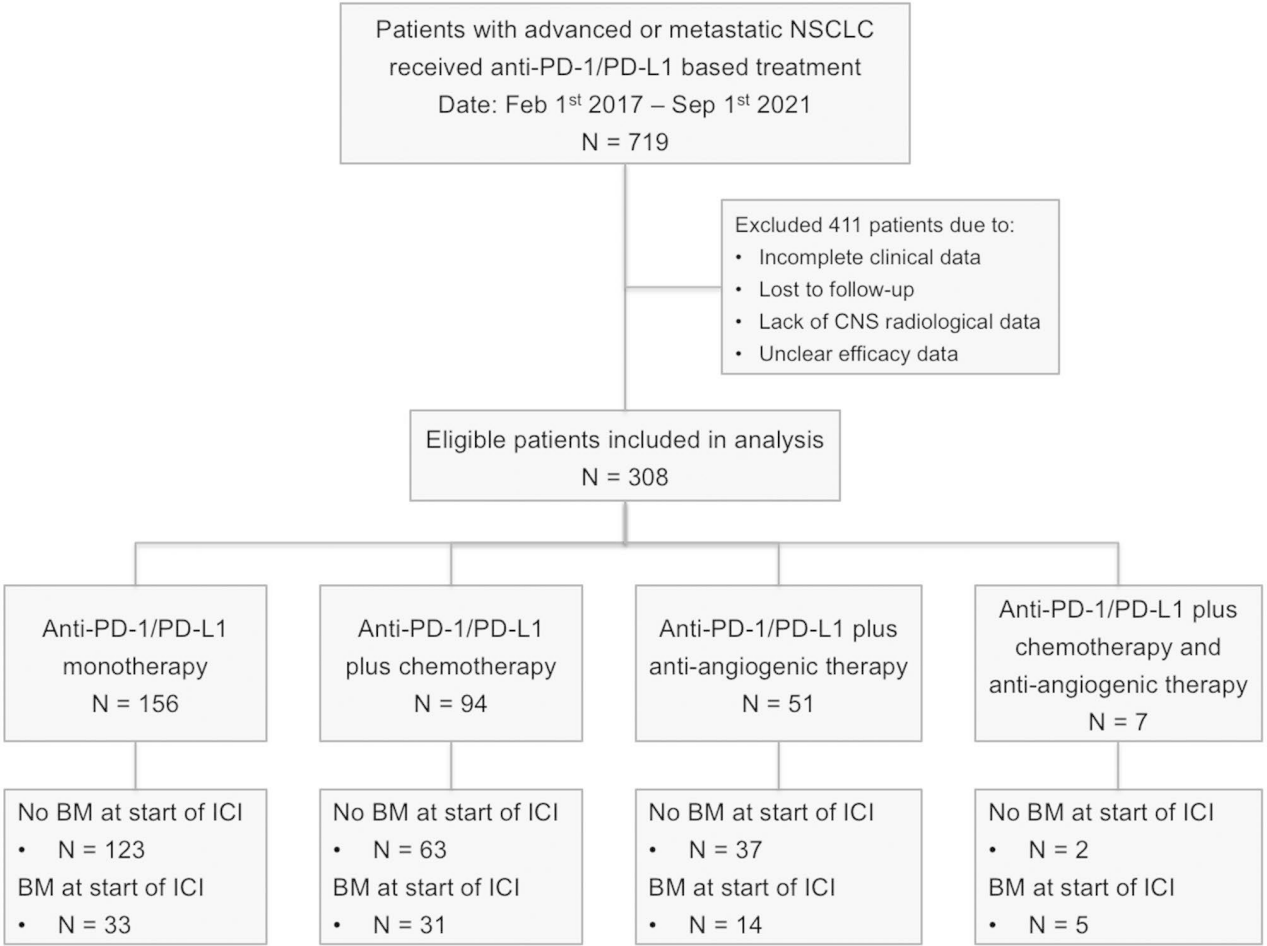
Flowchart of patients’ selection

719 patients with metastatic or advanced NSCLC treated with PD-1/PD-L1 inhibitor-based treatments were initially identified. 411 patients were excluded due to incomplete clinical data, lost to follow-up, lack of CNS radiological data, unclear efficacy data, etc. 308 cases were included in the final analysis (Fig. 1). The median age was 62 years (range, 20–89 years), 248 (79.9%) were male, 189 (61.4%) were smokers, 194 (63.0%) had histology of adenocarcinoma, 73 (23.7%) had driver gene alterations, 117 (38.0%) had positive PD-L1 expression, 269 (87.3%) had synchronous liver metastases, and 102 (33.1%) had corticosteroid usage history. 156 received anti-PD-1/PD-L1 monotherapy, 94 received anti-PD-1/PD-L1 plus chemotherapy, 51 received PD-1/PD-L1 inhibitor plus anti-angiogenic therapy and 7 received anti-PD-1/PD-L1 plus chemotherapy and anti-angiogenic therapy. 83 received treatment as first-line setting, 124 as second-line and 101 as third or above-line setting (Table 1). The overall objective response rate (ORR) was 26.3% and disease control rate (DCR) was 67.5%. With a median follow-up of 24.5 months (range, 5.2 to 47.9), the median PFS and OS was 4.1 and 11.9 months. Most of the baseline parameters including age, sex, smoking history, ECOG PS, histology, driver gene alteration rate and BrM-related symptoms at start of ICI were well balanced between patients received ICI monotherapy and ICI based combination therapy (Supplemental Table S1). Although patients treated with ICI monotherapy had markedly higher positive PD-L1 expression rate and less corticosteroid use before ICI than those treated with ICI based combination therapy, ORR (19.2% vs. 33.6%; *P* = 0.0043) and DCR (58.3% vs. 77.0%; *P* = 0.0005) were dramatically lower in ICI monotherapy than in ICI based combination therapy group (Supplemental Table S1).

**Table 1:** Baseline features of patients with or without brain metastasis

	Total (n = 308, %)	BrM (n = 83, %)	No BrM (n = 225, %)	*P* value
Median age, years (range)	62 (20–89)	58 (32–76)	63 (20–89)	
Sex				
Male	246 (79.9)	62 (74.7)	184 (81.8)	0.1692
Female	62 (20.1)	21 (25.3)	41 (18.2)	
Smoking history				
Current/former	189 (61.4)	45 (54.2)	144 (64.0)	0.1177
Never	119 (38.6)	38 (45.8)	81 (36.0)	
ECOG PS				
0–1	282 (91.6)	73 (88.0)	209 (92.9)	0.1667
2	26 (8.4)	10 (12.0)	16 (7.1)	
Pathology				
Adenocarcinoma	194 (63.0)	66 (79.5)	128 (56.9)	0.0003
Squamous carcinoma	79 (25.6)	9 (10.8)	70 (31.1)	
Others	35 (11.4)	8 (9.6)	27 (12.0)	
Driver gene alterations detection				
EGFR	28 (9.1)	12 (14.5)	16 (7.1)	
ALK/ROS1/RET	4 (1.3)	0 (0.0)	4 (1.8)	
KRAS	33 (10.7)	8 (9.6)	25 (11.1)	
BRAF	3 (1.0)	2 (2.4)	1 (0.4)	
HER2	5 (1.6)	2 (2.4)	3 (1.3)	
Wild type	137 (44.5)	41 (49.4)	96 (42.7)	0.7500
Unknown	98 (31.8)	18 (21.7)	80 (35.6)	
PD-L1 expression (IHC)				
Positive	117 (80.7)	21 (80.7)	96 (80.7)	0.7927
Negative	28 (19.3)	5 (19.3)	23 (19.3)	
Unknown	163	57	106	
Liver Metastasis				
Yes	269 (87.3)	69 (83.1)	200 (88.9)	0.1777
No	39 (12.7)	14 (16.9)	25 (11.1)	
Corticosteroid use before ICI				
Yes	102 (33.1)	27 (32.5)	75 (33.3)	0.8943
No	206 (66.9)	56 (67.5)	150 (66.7)	
Symptoms at start of ICI				
Asymptomatic BrM	-	50 (60.2)	-	
Symptomatic BrM	-	33 (39.8)	-	
Treatment strategy				
ICI alone	156 (50.6)	33 (39.8)	123 (54.7)	0.0202
ICI + chemotherapy	94 (30.5)	31 (37.3)	63 (28.0)	
ICI + anti-angiogenic therapy	51 (16.6)	14 (16.9)	37 (16.4)	
ICI + chemotherapy + anti-angiogenic therapy	7 (2.3)	5 (6.0)	2 (0.9)	
ICI treatment line				
1	83 (26.9)	21 (25.3)	62 (27.6)	0.6924
2	124 (40.3)	24 (28.9)	100 (44.4)	
3	64 (20.8)	19 (22.9)	45 (20.0)	
>3	37 (12.0)	19 (22.9)	18 (8.0)	
Systemic response				
PR	81 (26.3)	15 (18.1)	66 (29.3)	0.0464
SD	127 (41.2)	37 (44.6)	90 (40.0)	0.2664
PD	89 (28.9)	23 (27.7)	66 (29.3)	
NE	11 (3.6)	8 (9.6)	3 (1.3)	
BrM, brain metastasis; ECOG PS, Eastern Cooperative Oncology Group performance status; ICI, immune checkpoint inhibitor; IHC, immunohistochemistry; PR, partial response; SD, stable disease; PD, disease progression				

### The impact of BrMs on treatment outcomes

**Fig. 2:**
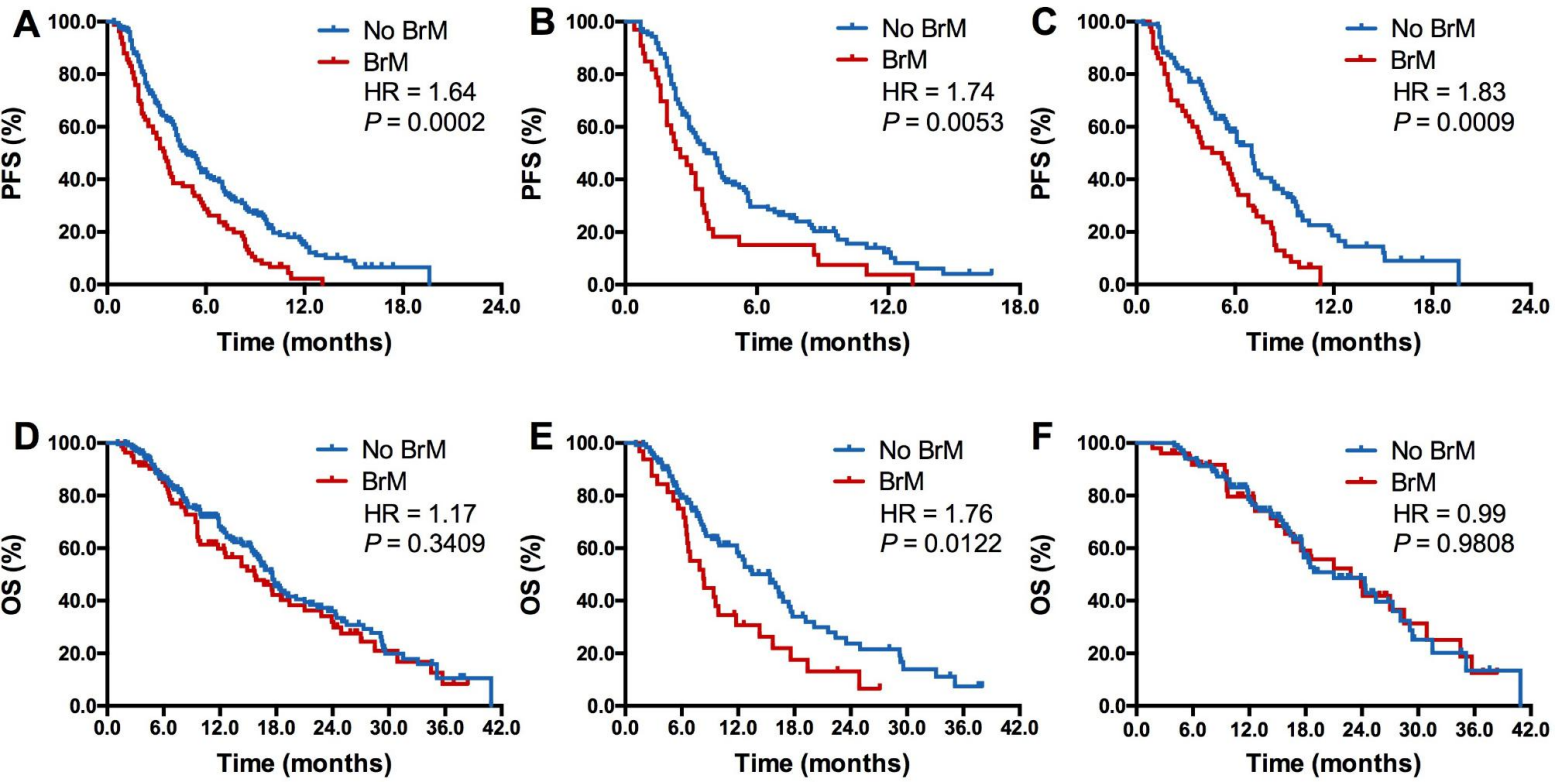
**Comparison of PFS and OS between patients with and without brain metastases**. (**A**), Comparison of PFS between patients with and without brain metastases in all included patients; (**B**), Comparison of PFS between patients with and without brain metastases in patients received ICI based monotherapy; (**C**), Comparison of PFS between patients with and without brain metastases in patients received ICI based combination therapy; (**D**), Comparison of OS between patients with and without brain metastases in all included patients; (**E**), Comparison of OS between patients with and without brain metastases in patients received ICI based monotherapy; (**F**), Comparison of OS between patients with and without brain metastases in patients received ICI based combination therapy. BrM, brain metastases

Compared with those without BrMs, patients with BrMs had significantly lower ORR (18.1% vs. 29.3%; *P* = 0.0464; Table 1) but similar DCR (62.7% vs. 69.3%; *P* = 0.2664; Table 1). The presence of BrMs was also associated with significantly inferior PFS (3.5 vs. 4.8 months; HR = 1.64, *P* = 0.0002; Fig. 2A) but comparable OS (15.7 vs. 17.6 months; HR = 1.17, *P* = 0.3409; Fig. 2D) in whole group. Subgroup analysis revealed that the presence of BrMs was correlated with both significantly inferior PFS (2.5 vs. 3.7 months; HR = 1.74, *P* = 0.0053; Fig. 2B) and OS (8.3 vs. 15.4 months; HR = 1.76, *P* = 0.0122; Fig. 2E) in patients received anti-PD-1/PD-L1 monotherapy. For those received PD-1/PD-L1 inhibitor-based combination therapy, the presence of BrMs was correlated with markedly shorter PFS (4.6 vs. 7.0 months; HR = 1.83, *P* = 0.0009; Fig. 2C) but similar OS (22.8 vs. 21.0 months; HR = 0.99, *P* = 0.9809; Fig. 2F). Intriguingly, we also observed the markedly different PFS but similar OS between patients with and without BrMs in anti-PD-1/PD-L1 plus chemotherapy group (Supplemental Figure S1A and Supplemental Figure S1B). However, both PFS and OS were analogous between those with and without BrMs in anti-PD-1/PD-L1 plus anti-angiogenic therapy group (Supplemental Figure S1C and Supplemental Figure S1D).

In all included cases, univariate analyses showed that female, smoking history, liver metastases, corticosteroid usage history, > 1 treatment line, ICIs monotherapy and BrMs were associated with significantly shorter PFS (Supplemental Table S2). Age ≥ 65, non-adenocarcinoma, liver metastases, negative PD-L1 expression, > 1 treatment line and ICIs monotherapy were associated with significantly inferior OS. Multivariate analyses showed that the presence of BrMs was independently correlated with shorter PFS (HR = 1.734, *P* < 0.001). In addition, liver metastases (HR = 1.585, *P* = 0.012) and ICIs monotherapy (HR = 1.597, *P* = 0.001) were also associated with shorter PFS (Supplemental Table S2). Only negative PD-L1 expression (HR = 1.404, *P* = 0.040) and ICIs monotherapy (HR = 1.842, *P* < 0.001) were correlated with shorter OS in multivariate analyses (Supplemental Table S2). In ICIs monotherapy group, the presence of BrMs was correlated with significantly inferior PFS and OS in multivariate analyses (Supplemental Table S3). However, in combination treatment, the presence of BrMs was correlated with poor PFS but similar OS in multivariate analyses (Supplemental Table S4).

### Baseline features of patients with BrMs

83 (26.9%) patients had BrMs. The median age was 58 years (range, 32–76 years), 62 (74.7%) were male, 45 (54.2%) were smokers, 66 (79.5%) had histology of adenocarcinoma, 24 (28.9%) had driver gene alterations, 21 (25.3%) had positive PD-L1 expression, 69 (83.1%) had synchronous liver metastases, 27 (32.5%) had corticosteroid usage history and 33 (39.8%) were symptomatic. 33 received anti-PD-1/PD-L1 monotherapy, 31 received anti-PD-1/PD-L1 plus chemotherapy, 14 received anti-PD-1/PD-L1 plus anti-angiogenic therapy and 5 received anti-PD-1/PD-L1 plus chemotherapy and anti-angiogenic therapy. 21 received treatment as first-line setting, 24 as second-line and 38 as third or above-line setting. Baseline features were summarized in Table 1. Compared with those without BrMs, patients with BrMs had more cases with history of adenocarcinoma (*P* = 0.0003) and received PD-1/PD-L1 inhibitor-based combination therapy (*P* = 0.0202).

### Treatment outcomes of patients with BrMs

**Fig. 3:**
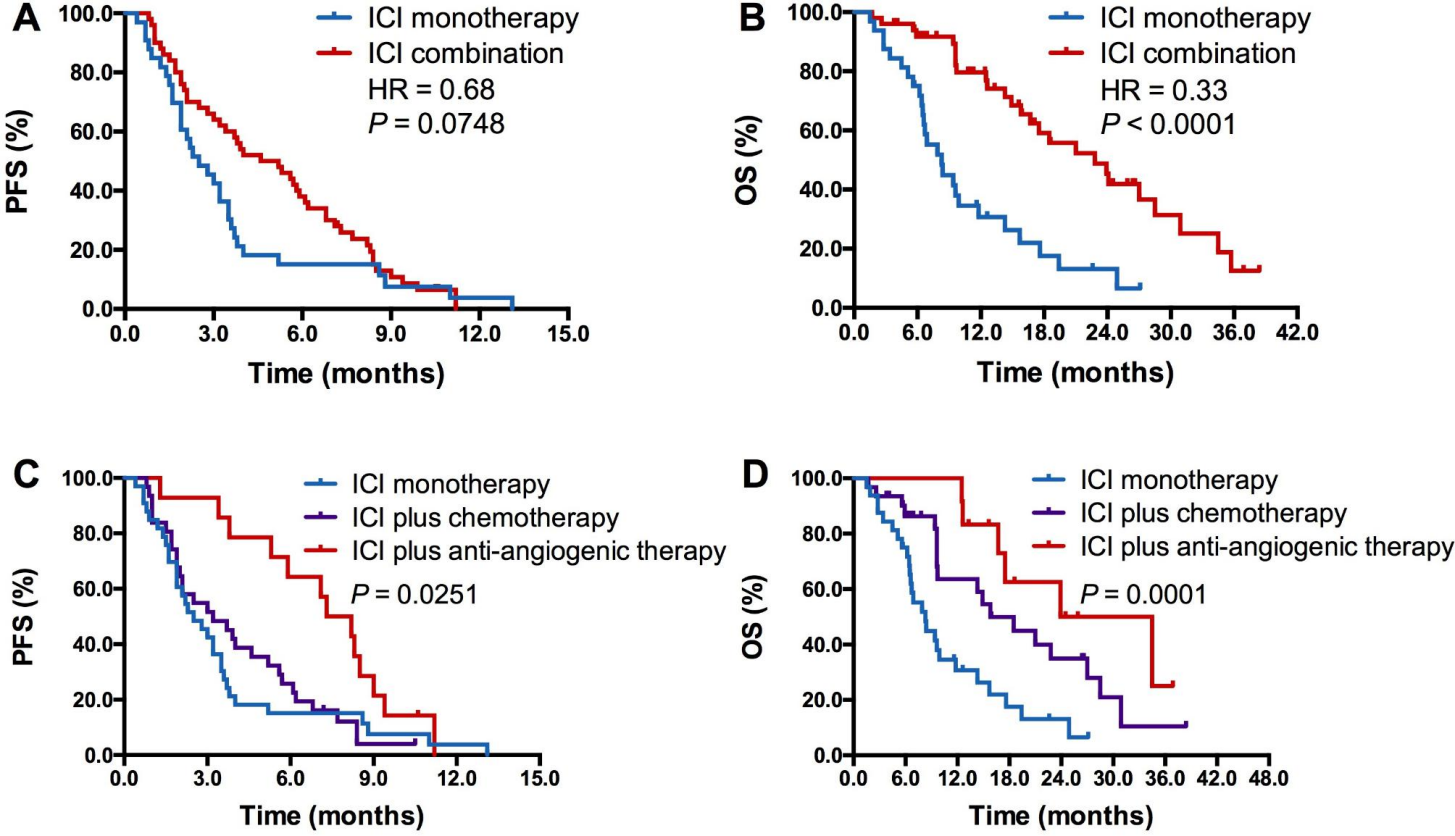
**Comparison of PFS and OS between ICI based monotherapy and combination therapy in patients with brain metastases**. (**A**), Comparison of PFS between ICI based monotherapy and combination therapy in patients with brain metastases; (**B**), Comparison of OS between ICI based monotherapy and combination therapy in patients with brain metastases; (**C**), Comparison of PFS among ICI based monotherapy, ICI plus chemotherapy and ICI plus anti-angiogenic therapy (including ICI plus chemotherapy and anti-angiogenic therapy) in patients with brain metastases; (**D**), Comparison of OS among ICI based monotherapy, ICI plus chemotherapy and ICI plus anti-angiogenic therapy (including ICI plus chemotherapy and anti-angiogenic therapy) in patients with brain metastasis

PD-1/PD-L1 inhibitor-based combination therapy was associated with prolonged PFS but it did not reach the statistical significance (4.6 vs. 2.5 months; HR = 0.68, *P* = 0.0748; Fig. 3A). However, patients with BrMs received combination therapy had significantly longer OS than those received monotherapy (22.8 vs. 8.3 months; HR = 0.33, *P* < 0.0001; Fig. 3B). Subgroup analyses suggested that anti-PD-1/PD-L1 plus antiangiogenic therapy (included those received anti-PD-1/PD-L1 plus chemotherapy and antiangiogenic therapy) had the longest PFS (7.7 vs. 3.2 vs. 2.5 months; *P* = 0.0251; Fig. 3C) and OS (29.2 vs. 15.8 vs. 8.3 months; *P* = 0.0001; Fig. 3D) when compared with anti-PD-1/PD-L1 plus chemotherapy and anti-PD-1/PD-L1 monotherapy. Univariate analyses showed that male, smoking history and PD-1/PD-L1 inhibitor-based combination therapy were correlated with longer PFS. Liver metastases and anti-PD-1/PD-L1 monotherapy were significantly correlated with shorter OS. In multivariate analyses, PD-1/PD-L1 inhibitor-based combination therapy was independently correlated with substantially prolonged PFS (HR = 0.586, *P* = 0.028) and OS (HR = 0.312, *P* < 0.001) (Supplemental Table S5).

Considering the potential impact of driver gene alterations and PD-L1 expression level on the efficacy of ICIs treatment in advanced NSCLC, we therefore conducted the subgroup analysis based on the driver gene status and PD-L1 expression in BrM group. We observed that BrM patients with one of common driver gene alterations (including *EGFR, HER2, BRAF, KRAS, ALK, ROS1, RET*) had similar PFS (HR = 1.40, *P* = 0.1879; Supplemental Figure S2A) and OS (HR = 1.16, *P* = 0.6438; Supplemental Figure S2B) to BrM patients without common driver gene alterations. Although BrM patients with one of common driver gene alterations received ICI based combination therapy had similar PFS (HR = 0.57, *P* = 0.1797; Supplemental Figure S2C) to those received ICI monotherapy, OS (HR = 0.27, *P* = 0.0070; Supplemental Figure S2D) was significantly longer in combination treatment group than monotherapy group, indicating that ICI based combination therapy should be considered for patients with driver gene alterations and BrMs. Interestingly, BrM patients with PD-L1 expression > 50% had both numerically better PFS (HR = 0.61, *P* = 0.1762; Supplemental Figure S3A) and OS (HR = 0.88, *P* = 0.7961; Supplemental Figure S3B) compared with those with PD-L1 expression ≤ 50%, mainly due to limited sample size.

### Characterization of TIME features of BrMs

**Fig. 4:**
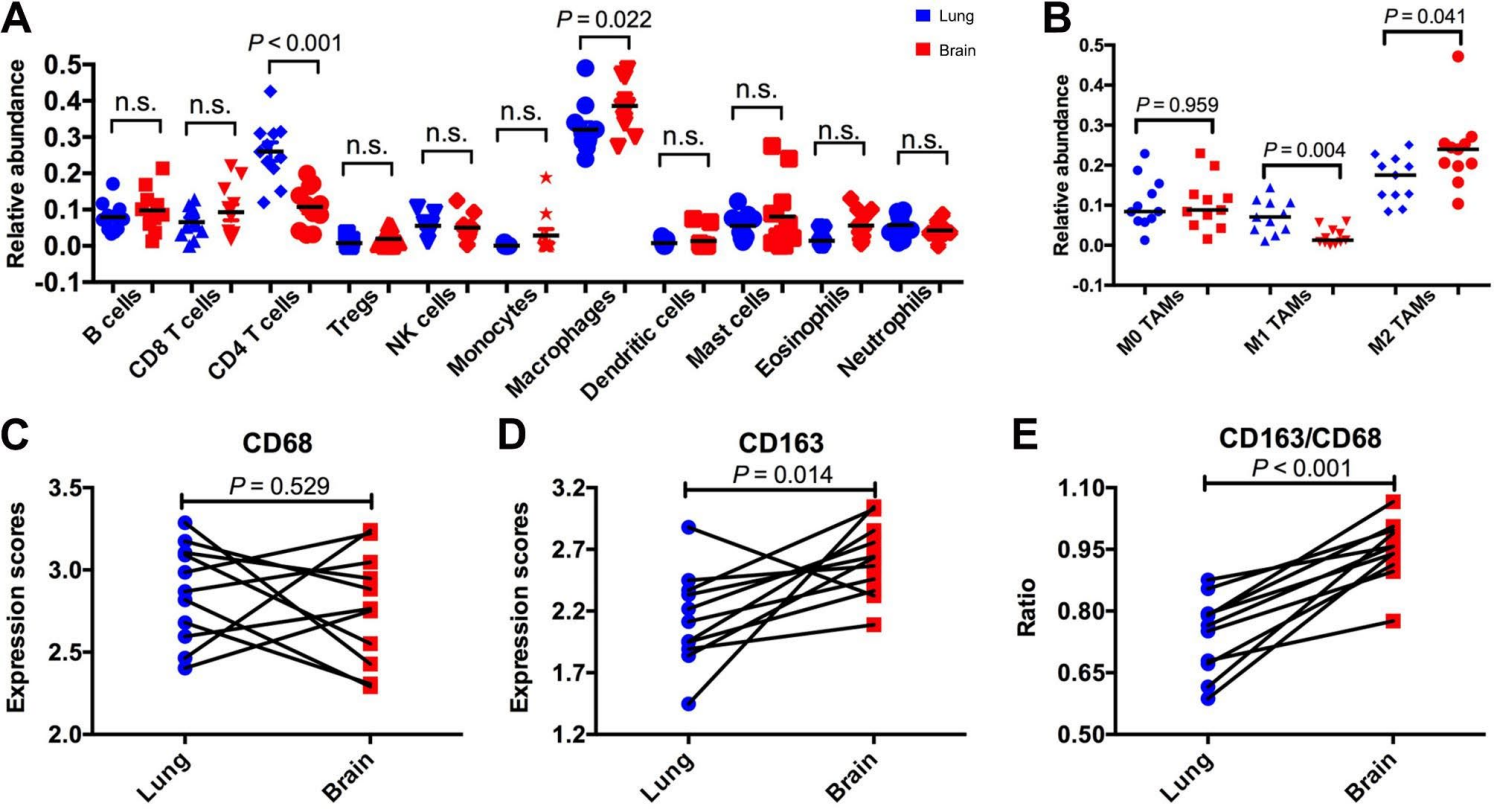
**Comparison of immune cell composition and the representative marker expression between paired primary lung tumors and brain metastases**. (**A**) 11 immune cell compositions comparison by using CIBERSORT between matched primary lung tumors and brain metastases. (**B**) Comparison of macrophage subtypes (M0, M1, M2) between matched primary lung tumors and brain metastases. (**C**) Comparison of CD68 expression level between matched primary lung tumors and brain metastases. (**D**) Comparison of CD163 expression level between matched primary lung tumors and brain metastases. (**E**) Comparison of CD163/CD68 expression level ratio between matched primary lung tumors and brain metastases

**Fig. 5:**
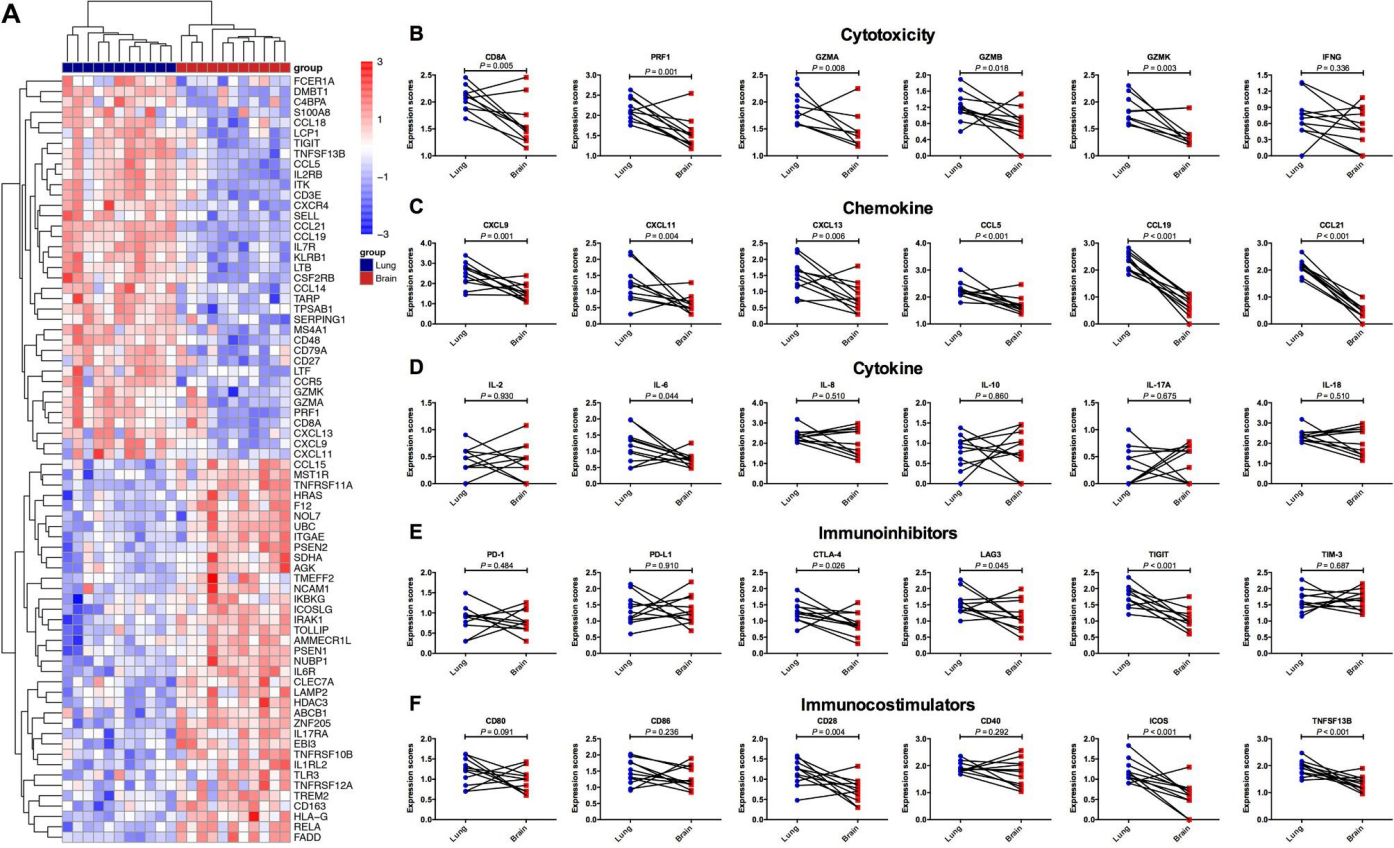
**Differentially gene expression analysis of matched primary lung tumors and brain metastases**. (**A**) Heatmap of differentially expression genes. (**B**) Representative marker expression level comparison of cytotoxicity between matched primary lung tumors and brain metastases. (**C**) Representative chemokine expression level comparison between matched primary lung tumors and brain metastases. (**D**) Representative cytokine expression level comparison between matched primary lung tumors and brain metastases. (**E**) Representative immunoinhibitors expression level comparison between matched primary lung tumors and brain metastases. (**F**) Representative immunocostimulators expression level comparison between matched primary lung tumors and brain metastases

To depict the specific TIME of BrMs and give the potential explanations for the above-mentioned distinct treatment outcomes, we analyzed the RNA-seq data of 22 samples from eleven paired primary lung cancers and BrMs. We first quantified the relative infiltration of several immune cell subtypes using CIBERSORT. As shown in Fig. 4A, lower fractions of CD4^+^ T cells, and higher fractions of macrophages were observed in BrMs. Subtypes analysis revealed that BrMs had significantly higher fractions of M2 macrophages but lower fractions of M1 macrophages than the matched primary lesions (Fig. 4B). Representative markers expression level of macrophages including CD68 and CD163 was consistent with this finding (Fig. 4C-E). Consistent with previous studies (17, 25, 26), differentially gene expression analysis showed that BrMs had dramatically decreased cytotoxicity (CD8A, PRF1, GZMA, GZMB, GZMK, IFNG), chemokine (CXCL9, CXCL11, CXCL13, CCL5, CCL19, CCL21), proinflammatory cytokine (IL-6), immunoinhibitors (CTLA-4, LAG3, TIGIT) and immunocostimulators (CD28, ICOS, TNFSF13B), compared with primary lung cancers (Fig. 5A-F and Supplemental Figure S4), suggesting a suppressed TIME in BrMs. The mRNA expression level of PD-1 and PD-L1 was similar between paired primary lung cancers and BrMs (Fig. 5E).

## Discussion

To date, PD-1/PD-L1 inhibitors monotherapy or plus chemotherapy have become the new standard of care for patients with NSCLC without driver gene alterations in first-line setting. Nonetheless, the optimal treatment strategy for patients with NSCLC and BrMs remains undetermined mainly due to lack of clear immunophenotyping of BrMs. Previous publications on these populations reported heterogeneous results. Here, the current study enrolled 308 patients and reported that BrMs presence was correlated with significantly shorter PFS and OS in anti-PD-1/PD-L1 monotherapy group, while it was only associated with inferior PFS but similar OS in anti-PD-1/PD-L1 based combination treatment group. Of patients with BrMs, anti-PD-1/PD-L1 plus antiangiogenic therapy was associated with the longest PFS and OS when compared with anti-PD-1/PD-L1 monotherapy or plus chemotherapy. Multivariate analyses showed that anti-PD-1/PD-L1 based combination treatment was independently correlated with significantly longer PFS and OS in patients with BrMs. Furthermore, transcriptomic analysis of paired primary lung tumors and BrMs showed a suppressive TIME in BrMs with decreased CD4^+^ T cells and M1 macrophages but increased M2 macrophages infiltration. Collectively, these findings indicate that BrMs of NSCLC possessed an immunosuppressive TIME; anti-PD-1/PD-L1 monotherapy showed limited antitumor efficacy in patients with NSCLC and BrMs; the anti-PD-1/PD-L1 based combination treatment, especially anti-PD-1/PD-L1 plus anti-angiogenic treatment, could be an alternative and effective treatment option for patients with NSCLC and BrMs.

In this study, 308 patients with anti-PD-1/PD-L1 treated metastatic or advanced NSCLC were included and 83 (26.9%) of them had BrMs. Although the percentage is higher than that reported in previous studies [16, 22, 23], it was similar to that reported in a series of retrospective studies [12, 24, 25] and is expected in this population. Of note, the rate of patients with corticosteroid usage history (33.1%) was much higher than that in a recent study [12] mainly due to the high percentage of patients with symptomatic BrMs (39.8%) in our study.

BrM is one of the negative prognostic factors in patients with NSCLC [26]. The efficacy of anti-PD-1/PD-L1 based treatment in patients with BrMs remained undetermined. In the current study, despite much more cases with histology of adenocarcinoma and received ICIs-based combination therapy, the overall ORR was significantly lower in patients with BrMs than those without BrMs (18.1% vs. 29.3%; *P* = 0.0464). Moreover, we observed that the presence of BrMs was correlated with significantly inferior PFS and OS in anti-PD-1/PD-L1 monotherapy group. These results were consistent with the findings of the French expanded access program series [11]. However, a recent retrospective study included 1025 patients showed that the ORR was comparable between patients with and without BrMs (20.6% vs. 22.7%) and the presence of BrMs was not correlated with inferior survival with ICI monotherapy in multivariate analysis [12]. The potential reason for this discordance may include the different baseline features and ethnicity of research populations, high percentage of patients with symptomatic BrMs, and high rate of patients with liver metastasis in this study. Collectively, BrMs presence showed limited impact on the efficacy of anti-PD-1/PD-L1based combination therapy but the impact of BrMs presence on efficacy of anti-PD-1/PD-L1monotherapy in NSCLC still remains undetermined. Future prospective studies with larger populations and strict design are warranted.

Several clinical trials suggested that PD-1/PD-L1 inhibitor plus chemotherapy and/or antiangiogenic therapy could dramatically prolong both PFS and OS in patients with NSCLC [13–15]. Furthermore, the updated analysis from KEYNOTE-189 showed that pembrolizumab plus chemotherapy could also benefit patients with BrMs, which reported that HRs for OS and PFS were comparable regardless of BrMs [16]. Consistently, our study found that patients received anti-PD-1/PD-L1 based combination therapy had substantially longer PFS and OS than those received anti-PD-1/PD-L1 monotherapy. Subgroup analysis showed that PD-1/PD-L1 inhibitor plus antiangiogenic therapy was correlated with the longest PFS and OS when compared with PD-1/PD-L1 inhibitor plus chemotherapy and anti-PD-1/PD-L1 monotherapy. Recently, Kudo et al. reported that TIME of BrMs from NSCLC is immunosuppressed with less T cell infiltration and increased immunosuppressive tumor-associated macrophages [27]. Li et al. also reported that BrMs of lung cancers had reduced tumor infiltrating lymphocytes, decreased scores of immune-related signatures, and a lower proportion of tumors with high PD-L1/high CD8A [28]. Our current results were consistent with these two studies. In addition, the current findings showed that BrMs had an increased infiltration of M2 macrophages. Our previous study reported that reasonable dose of antiangiogenic agents could induce the polarization of M2 tumor-associated macrophages to M1 tumor-associated macrophages in TIME of lung cancer, subsequently potentiating the antitumor effect of PD-1/PD-L1 inhibitors [29]. Thus, we could hypothesize that the addition of antiangiogenic drug increased the antitumor effect of PD-1/PD-L1 inhibitor via shaping the phenotype of tumor-associated macrophage in BrMs. Collectively, these findings indicate that PD-1/PD-L1 inhibitor plus antiangiogenic therapy may be one of the promising therapeutic options for patients with NSCLC and BrMs. Notably, the innovative clinical trial to investigate the safety and efficacy of bevacizumab plus pembrolizumab in patients with melanoma and NSCLC BrMs is also ongoing (NCT02681549) and the results are anticipated.

Previous publications revealed that common driver gene alterations and low or negative PD-L1 expression level could impair the efficacy of ICIs treatment in advanced NSCLC [30–32]. Hence, we conducted the subgroup analysis based on the driver gene status and PD-L1 expression in patients with BrMs. The results showed that BrM patients with one of common driver gene alterations received ICI based combination therapy had significantly longer OS to those received ICI monotherapy, indicating that patients with driver gene alterations and BrMs could be treated with ICI based combination therapy. Interestingly, BrM patients with PD-L1 expression > 50% had both numerically better PFS and OS compared with those with PD-L1 expression ≤ 50%, suggesting PD-L1 expression may have potential impact on the efficacy of ICIs treatment in advanced NSCLC with BrMs. This is reminiscent of the updated analysis of a phase II trial that reported intracranial ORR of 27.3% in PD-L1-positive patients with NSCLC and untreated BrMs received pembrolizumab, while no BrM response was observed in PD-L1-negative cohort [10], suggesting the importance of appropriate biomarker selection in treatment decision for this population.

The current study had several limitations that should be acknowledged. First, the small sample size and the retrospective nature will inevitably have several biases such as selection bias. Second, the cranial radiotherapy history was not recorded in details, resulting in the bias of outcomes assessment. Given the radiotherapy could not only alter the immune microenvironment of BrMs but also enhance the efficacy of ICI through synergy effect or abscopal effect, we should emphasize that these results should be interpreted with caution and large-scale prospective study is still warranted. Intriguingly, Lizza et al. reported that cranial radiotherapy before start of ICIs treatment was not correlated with OS in NSCLC with BrMs [12], suggesting its uncertain effect on ICIs treatment for BrMs disease. Third, we did not record the intracranial tumor response and number and disease status of BrMs (active or stable), making further subgroup analysis difficult. Fourth, although previous studies revealed the different efficacy of PD-1 and PD-L1 inhibitors in solid tumors [33], we did not investigate its potential impact on the combination therapy due to limited sample size. Last but not least, we did not evaluate several potential biomarkers including tumor mutational burden, pro-inflammatory gene signatures, etc. due to limited tissue samples. Further investigation of these biomarkers is required to investigate their predictive or prognostic value in patients with NSCLC and BrMs.

In summary, the current study suggests that NSCLC with BrMs could obtain barely satisfactory treatment benefit from anti-PD-1/PD-L1 monotherapy, partly due to the specific immunosuppressive TIME of BrMs. Anti-PD-1/PD-L1 based combination therapy could significantly improve the clinical outcomes of patients with NSCLC and BrMs. Particularly, anti-PD-1/PD-L1 plus anti-angiogenic treatment was correlated with the longest PFS and OS, indicating that this combination strategy might be one of the promising therapeutic options for these populations. Given the retrospective nature and small sample size of this study, future large-scale prospective study is warranted to validate the current findings.

## Electronic supplementary material

Below is the link to the electronic supplementary material.

Supplementary Material 1

## Data Availability

The datasets used and/or analyzed during the current study are available from the corresponding author on reasonable request.

## Abbreviations

BrMs: Brain metastases
CI: Confidence interval
CR: Complete response
CT: Computed tomography
DCR: Disease control rate
ECOG PS: Eastern Cooperative Oncology Group performance status
HR: Hazard ratios
ICIs: Immune-checkpoint inhibitors
LUAD: Lung adenocarcinoma
MRI: Magnetic resonance imaging
NSCLC: Non-small-cell lung cancer
ORR: Objective response rate
OS: Overall survival
PD: Progressive disease
PD-1: Programmed cell death 1
PD-L1: Programmed cell death ligand 1
PET: Positron emission tomography
PR: Partial response
SD: Stable disease
TIME: Tumor immune microenvironment
