# Supplementary material for: Outcome differences between PD-1/PD-L1 inhibitors-based monotherapy and combination treatments in NSCLC with brain metastases
Source: Exp Hematol Oncol. 2023 Jun 23;12:56. doi: 10.1186/s40164-023-00412-3 (PMC10288673; doi:10.1186/s40164-023-00412-3)
Supplement: Supplementary file 1 — Supplementary Material 1 [file 40164_2023_412_MOESM1_ESM.docx]

**Supplemental Material**

**Outcome differences between PD-1/PD-L1 inhibitors based monotherapy and combination therapy in non-small-cell lung cancer with brain metastases**

Haowei Wang, Fangfang Liu, Xiaoxia Chen, Chao Zhao, Xuefei Li, Caicun Zhou, Jie Hu, Qian Chu, Tao Jiang

Supplemental Figure S1……………………………………………………….………..2

Supplemental Figure S2……………………………………………………….………..3

Supplemental Figure S3……………………………………………………….………..4

Supplemental Figure S4……………………………………………………….………..5

Supplemental Table S1……………………………………………………………….…6

Supplemental Table S2……………………………………………………………….…9

Supplemental Table S3…………………………………………………………………11

Supplemental Table S4…………………………………………………………………13

Supplemental Table S5…………………………………………………………………15


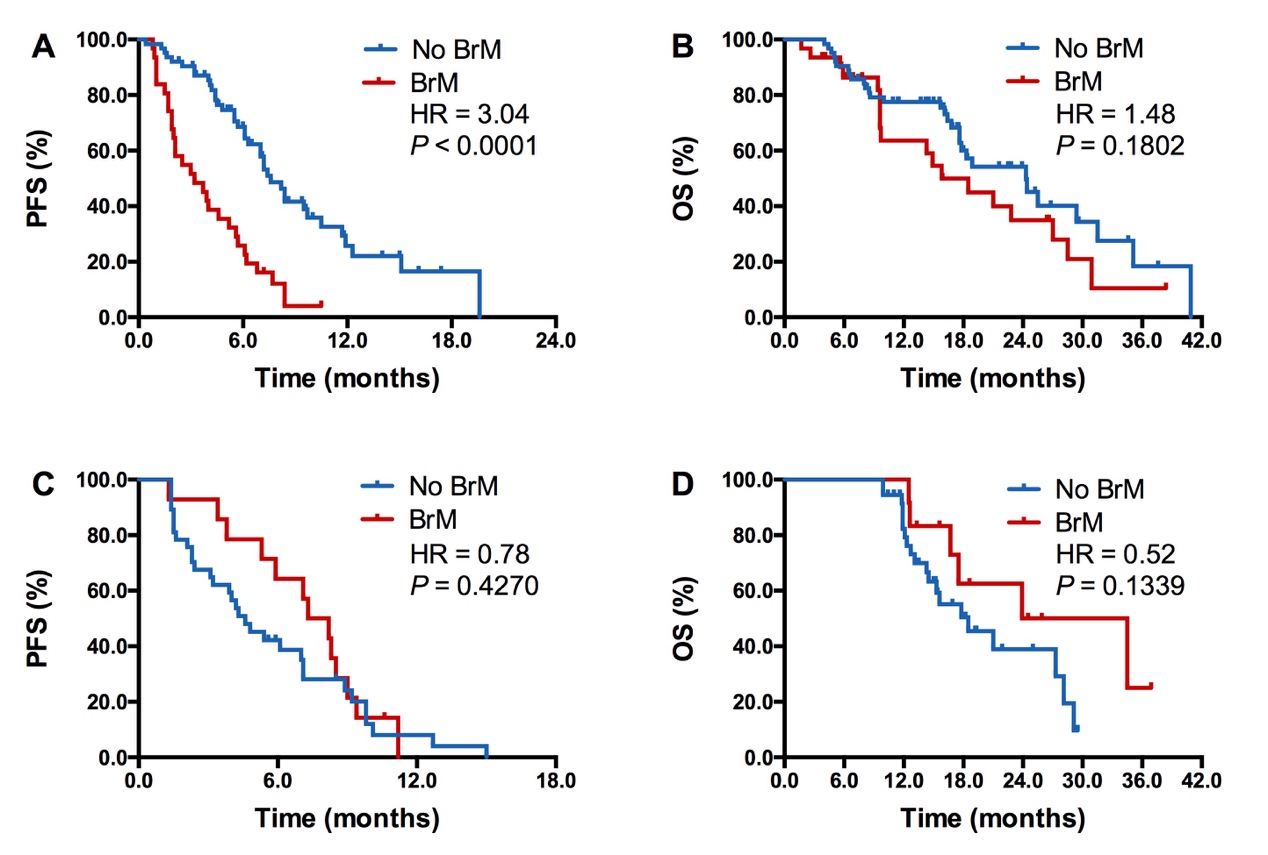


**Supplemental Figure S1.** **Comparison of PFS and OS between patients with and without brain metastases. A,** Comparison of PFS between patients with and without brain metastases in patients received anti-PD-1/PD-L1 antibodies plus chemotherapy; **B**, Comparison of OS between patients with and without brain metastases in patients received anti-PD-1/PD-L1 antibodies plus chemotherapy; **C,** Comparison of PFS between patients with and without brain metastases in patients received anti-PD-1/PD-L1 antibodies plus chemotherapy and anti-angiogenic therapy; **D**, Comparison of OS between patients with and without brain metastases in patients received anti-PD-1/PD-L1 antibodies plus chemotherapy and anti-angiogenic therapy. BrM, brain metastases.


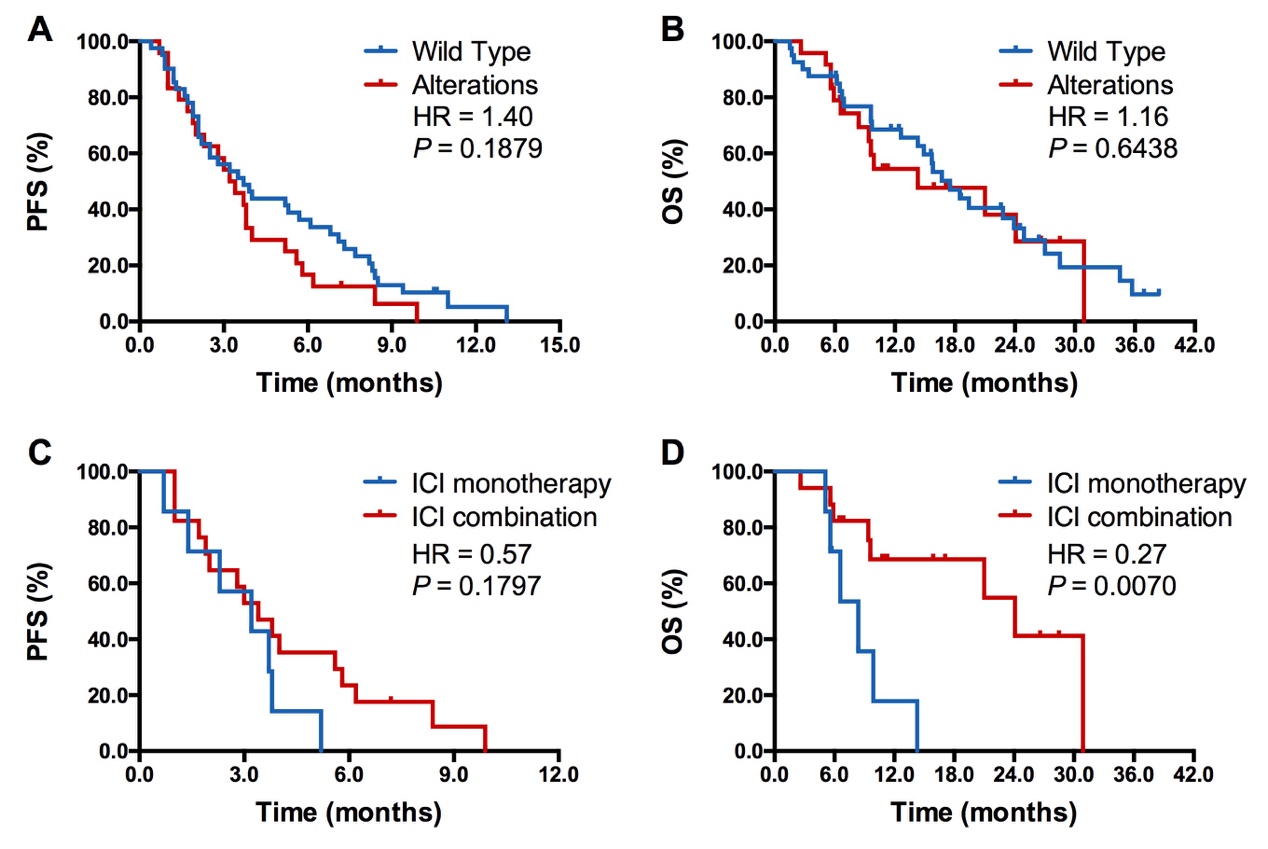


**Supplemental Figure S2.** **Subgroup analysis in patients with BrM and driver gene alterations. A,** Comparison of PFS between patients with and without driver gene alterations in BrM group; **B**, Comparison of OS between patients with and without driver gene alterations in BrM group; **C,** Comparison of PFS between ICI monotherapy and combination therapy group in patients with BrM and driver gene alterations; **D**, Comparison of OS between ICI monotherapy and combination therapy group in patients with BrM and driver gene alterations. BrM, brain metastases.


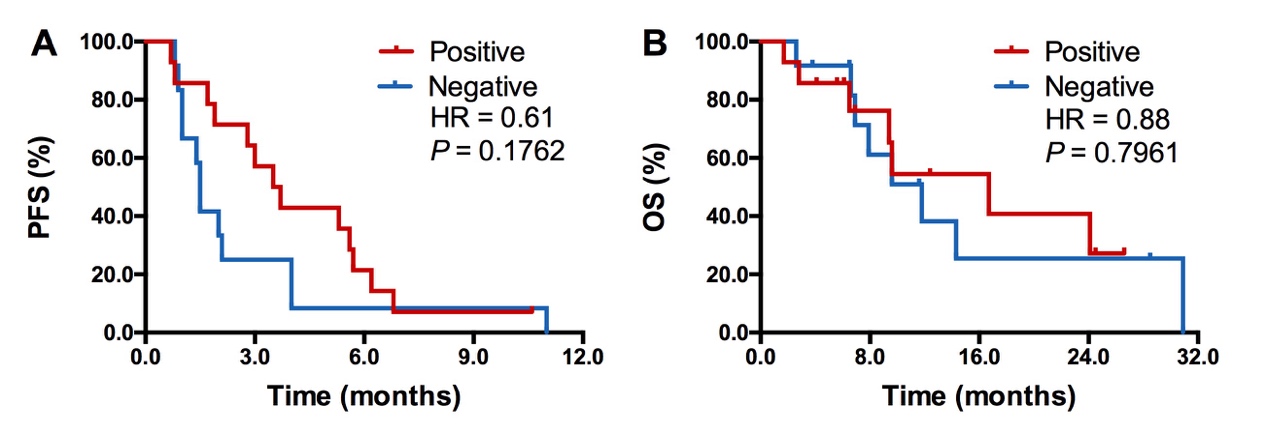


**Supplemental Figure S3.** **Subgroup analysis in patients with BrM and PD-L1 expression result. A,** Comparison of PFS between patients with and without high PD-L1 expression (cutoff = 50%) in BrM group; **B**, Comparison of OS between patients with and without high PD-L1 expression (cutoff = 50%) in BrM group. BrM, brain metastases.


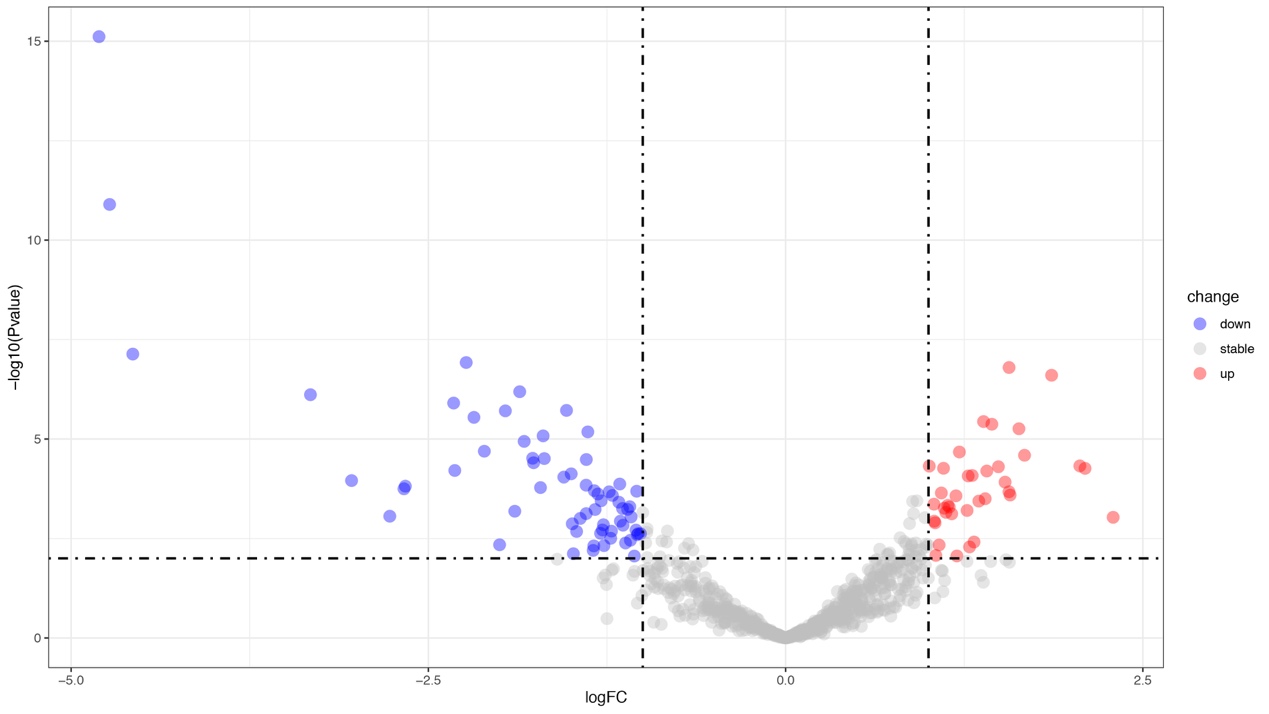


**Supplemental Figure S4.** **Volcano plot showed all of the differential expression genes between eleven paired primary lung cancers and brain metastasis.**

| **Supplemental Table S1. Baseline features of patients received ICI based monotherapy versus combination therapy.** | | | | |
| --- | --- | --- | --- | --- |
|  | **Total (n=308, %)** | **ICI monotherapy (n=156, %)** | **ICI combination therapy (n=152, %)** | ***P* value** |
| Median age, years (range) | 62 (20-89) | 64 (29-89) | 60 (20-80) |  |
| Sex |  |  |  |  |
| Male | 246 (79.9) | 126 (80.8) | 120 (78.9) | 0.6901 |
| Female | 62 (20.1) | 30 (19.2) | 32 (21.1) |  |
| Smoking history |  |  |  |  |
| Current/former | 189 (61.4) | 103 (66.0) | 86 (56.6) | 0.0887 |
| Never | 119 (38.6) | 53 (34.0) | 66 (43.4) |  |
| ECOG PS |  |  |  |  |
| 0-1 | 282 (91.6) | 146 (93.6) | 136 (89.5) | 0.1939 |
| 2 | 26 (8.4) | 10 (6.4) | 16 (10.5) |  |
| Pathology |  |  |  |  |
| Adenocarcinoma | 194 (63.0) | 75 (48.1) | 119 (78.3) |  |
| Squamous carcinoma | 79 (25.6) | 63 (40.4) | 16 (10.5) |  |
| Others | 35 (11.4) | 18 (11.5) | 17 (11.2) | 0.9220 |
| Driver gene alterations detection |  |  |  |  |
| EGFR | 28 (9.1) | 8 (5.1) | 20 (13.2) |  |
| ALK/ROS1/RET | 4 (1.3) | 1 (0.6） | 3 (2.0) |  |
| KRAS | 33 (10.7) | 17 (10.9) | 16 (10.5) |  |
| BRAF | 3 (1.0) | 1 (0.6) | 2 (1.3) |  |
| HER2 | 5 (1.6) | 0 (0.0) | 5 (3.3) |  |
| Wild type | 137 (44.5) | 69 (44.2) | 68 (44.7) | 0.9288 |
| Unknown | 98 (31.8) | 60 (38.6) | 38 (25.0) |  |
| PD-L1 expression (IHC) |  |  |  |  |
| Positive | 117 (80.7) | 66 (88.0) | 51 (72.9) | 0.0210 |
| Negative | 28 (19.3) | 9 (12.0) | 19 (27.1) |  |
| Unknown | 163 | 81 | 82 |  |
| Liver Metastasis |  |  |  |  |
| Yes | 269 (87.3) | 134 (85.9) | 135 (88.8) | 0.4413 |
| No | 39 (12.7) | 22 (14.1) | 17 (11.2) |  |
| Corticosteroid use before ICI |  |  |  |  |
| Yes | 102 (33.1) | 41 (26.3) | 61 (40.1) | 0.0098 |
| No | 206 (66.9) | 115 (73.7) | 91 (59.9) |  |
| BrM before ICI based therapy |  |  |  |  |
| Yes | 83 (26.9) | 33 (21.2) | 50 (32.9) | 0.0202 |
| No | 225 (73.1) | 123 (78.8) | 102 (67.1) |  |
| Symptoms at start of ICI |  |  |  |  |
| Asymptomatic BrM | 50 (60.2) | 23 (69.7) | 27 (54.0) | 0.1527 |
| Symptomatic BrM | 33 (39.8) | 10 (30.3) | 23 (46.0) |  |
| ICI treatment line |  |  |  |  |
| 1 | 83 (26.9) | 21 (13.5) | 62 (40.8) | <0.0001 |
| 2 | 124 (40.3) | 83 (53.2) | 41 (27.0) |  |
| 3 | 64 (20.8) | 28 (17.9) | 36 (23.7) |  |
| >3 | 37 (12.0) | 24 (15.4) | 13 (8.5) |  |
| Systemic response |  |  |  |  |
| PR | 81 (26.3) | 30 (19.2) | 51 (33.6) | 0.0043 |
| SD | 127 (41.2) | 61 (39.1) | 66 (43.4) | 0.0005 |
| PD | 89 (28.9) | 63 (40.4) | 26 (17.1) |  |
| NE | 11 (3.6) | 2 (1.3) | 9 (5.9) |  |
| BrM, brain metastasis; ECOG PS,Eastern Cooperative Oncology Group performance status; ICI, immune checkpoint inhibitor; IHC, immunohistochemistry; PR, partial response; SD, stable disease; PD, disease progression. | | | | |

| **Supplemental Table S2. Univariate and multivariate analyses of clinical parameters on progression-free survival and overall survival in all included patients.** | | | | | | | |
| --- | --- | --- | --- | --- | --- | --- | --- |
| **Factor** | **Univariate analysis** | | |  | **Multivariate analysis** | | |
|  | **HR (log rank)** | **95% CI** | ***P* value** |  | **HR (log rank)** | **95% CI** | ***P* value** |
| ***Progression-free survival*** |  |  |  |  |  |  |  |
| Sex (Female/male) | 1.368 | 1.005-1.862 | 0.047 |  | 1.117 | 0.749-1.664 | 0.588 |
| Age (≥65/<65) | 1.202 | 0.929-1.556 | 0.162 |  |  |  |  |
| Smoking (yes/no) | 0.771 | 0.595-1.000 | 0.050 |  | 0.788 | 0.560-1.108 | 0.110 |
| ECOG PS (>1/0) | 1.377 | 0.871-2.177 | 0.171 |  |  |  |  |
| Histology (adeno/non-adeno) | 0.887 | 0.684-1.151 | 0.369 |  |  |  |  |
| Brain metastasis (yes/no) | 1.639 | 1.322-2.406 | <0.001 |  | 1.734 | 1.315-2.287 | <0.001 |
| Liver metastasis (yes/no) | 1.923 | 1.357-2.725 | <0.001 |  | 1.585 | 1.106-2.271 | 0.012 |
| PD-L1 expression (yes/no or unknown) | 0.901 | 0.696-1.166 | 0.428 |  |  |  |  |
| Driver gene mutation (yes/no or unknown) | 1.040 | 0.770-1.405 | 0.797 |  |  |  |  |
| Corticosteroid use before ICI (yes/no) | 1.267 | 0.971-1.655 | 0.082 |  | 1.298 | 0.985-1.710 | 0.064 |
| Treatment line (1/>1) | 0.636 | 0.473-0.855 | 0.003 |  | 0.776 | 0.566-1.063 | 0.114 |
| Treatment (ICI monotherapy/ICI combination) | 1.511 | 1.175-1.943 | 0.001 |  | 1.597 | 1.210-2.108 | 0.001 |
| ***Overall survival*** |  |  |  |  |  |  |  |
| Sex (Female/male) | 1.088 | 0.750-1.579 | 0.655 |  |  |  |  |
| Age (≥65/<65) | 1.357 | 1.001-1.840 | 0.049 |  | 1.270 | 0.927-1.741 | 0.137 |
| Smoking (yes/no) | 0.848 | 0.625-1.152 | 0.292 |  |  |  |  |
| ECOG PS (>1/0) | 1.590 | 0.883-2.860 | 0.122 |  |  |  |  |
| Histology (adeno/non-adeno) | 0.685 | 0.503-0.933 | 0.016 |  | 0.869 | 0.626-1.204 | 0.398 |
| Brain metastasis (yes/no) | 1.170 | 0.842-1.651 | 0.341 |  |  |  |  |
| Liver metastasis (yes/no) | 1.455 | 0.970-2.183 | 0.070 |  | 1.473 | 0.979-2.217 | 0.063 |
| PD-L1 expression (yes/no) | 0.692 | 0.502-0.953 | 0.024 |  | 0.712 | 0.515-0.984 | 0.040 |
| Driver gene mutation (yes/no) | 1.122 | 0.779-1.616 | 0.536 |  |  |  |  |
| Corticosteroid use before ICI (yes/no) | 1.106 | 0.806-1.517 | 0.532 |  |  |  |  |
| Treatment line (1/>1) | 0.614 | 0.425-0.887 | 0.009 |  | 0.749 | 0.510-1.100 | 0.141 |
| Treatment (ICI monotherapy/ICI combination) | 2.071 | 1.528-2.808 | <0.001 |  | 1.842 | 1.326-2.560 | <0.001 |
| HR: hazard ratio; CI: confidence interval; ECOG PS,Eastern Cooperative Oncology Group performance status; ICI, immune checkpoint inhibitor; No., number. | | | | | | | |

| **Supplemental Table S3. Univariate and multivariate analyses of clinical parameters on progression-free survival and overall survival in patients received ICI based monotherapy.** | | | | | | | |
| --- | --- | --- | --- | --- | --- | --- | --- |
| **Factor** | **Univariate analysis** | | |  | **Multivariate analysis** | | |
|  | **HR (log rank)** | **95% CI** | ***P* value** |  | **HR (log rank)** | **95% CI** | ***P* value** |
| ***Progression-free survival*** |  |  |  |  |  |  |  |
| Sex (Female/male) | 0.753 | 0.486-1.167 | 0.205 |  |  |  |  |
| Age (≥65/<65) | 1.109 | 0.784-1.570 | 0.559 |  |  |  |  |
| Smoking (yes/no) | 0.705 | 0.485-1.026 | 0.068 |  | 0.711 | 0.487-1.037 | 0.076 |
| ECOG PS (>1/0) | 1.096 | 0.447-2.688 | 0.840 |  |  |  |  |
| Histology (adeno/non-adeno) | 0.974 | 0.688-1.380 | 0.883 |  |  |  |  |
| Brain metastasis (yes/no) | 1.746 | 1.168-2.610 | 0.007 |  | 1.614 | 1.058-2.462 | 0.026 |
| Liver metastasis (yes/no) | 1.628 | 1.026-2582 | 0.039 |  | 1.396 | 0.860-2.268 | 0.177 |
| PD-L1 expression (yes/no or unknown) | 0.864 | 0.602-1.240 | 0.428 |  |  |  |  |
| Driver gene mutation (yes/no or unknown) | 1.076 | 0.667-1.736 | 0.763 |  |  |  |  |
| Corticosteroid use before ICI (yes/no) | 1.193 | 0.801-1.776 | 0.385 |  |  |  |  |
| Treatment line (1/>1) | 0.887 | 0.531-1.480 | 0.645 |  |  |  |  |
| ***Overall survival*** |  |  |  |  |  |  |  |
| Sex (Female/male) | 0.993 | 0.593-1.661 | 0.978 |  |  |  |  |
| Age (≥65/<65) | 1.398 | 0.929-2.104 | 0.108 |  |  |  |  |
| Smoking (yes/no) | 0.935 | 0.606-1.443 | 0.761 |  |  |  |  |
| ECOG PS (>1/0) | 1.894 | 0.763-4.717 | 0.169 |  |  |  |  |
| Histology (adeno/non-adeno) | 1.314 | 0.874-1.974 | 0.189 |  |  |  |  |
| Brain metastasis (yes/no) | 1.792 | 1.135-2.829 | 0.012 |  | 1.653 | 1.012-2.700 | 0.045 |
| Liver metastasis (yes/no) | 1.608 | 0.948-2.729 | 0.078 |  | 1.315 | 0.745-2.319 | 0.345 |
| PD-L1 expression (yes/no) | 0.942 | 0.609-1.458 | 0.788 |  |  |  |  |
| Driver gene mutation (yes/no) | 1.008 | 0.588-1.727 | 0.976 |  |  |  |  |
| Corticosteroid use before ICI (yes/no) | 1.249 | 0.803-1.943 | 0.324 |  |  |  |  |
| Treatment line (1/>1) | 0.797 | 0.424-1.497 | 0.480 |  |  |  |  |
| HR: hazard ratio; CI: confidence interval; ECOG PS, Eastern Cooperative Oncology Group performance status; ICI, immune checkpoint inhibitor; No., number. | | | | | | | |

| **Supplemental Table S4. Univariate and multivariate analyses of clinical parameters on progression-free survival and overall survival in patients received ICI based combination therapy.** | | | | | | | |
| --- | --- | --- | --- | --- | --- | --- | --- |
| **Factor** | **Univariate analysis** | | |  | **Multivariate analysis** | | |
|  | **HR (log rank)** | **95% CI** | ***P* value** |  | **HR (log rank)** | **95% CI** | ***P* value** |
| ***Progression-free survival*** |  |  |  |  |  |  |  |
| Sex (Female/male) | 0.731 | 0.472-1.134 | 0.162 |  |  |  |  |
| Age (≥65/<65) | 1.522 | 1.024-2.262 | 0.038 |  | 1.212 | 0.795-1.848 | 0.371 |
| Smoking (yes/no) | 0.773 | 0.535-1.117 | 0.171 |  |  |  |  |
| ECOG PS (>1/0) | 1.335 | 0.774-2`.3102 | 0.298 |  |  |  |  |
| Histology (adeno/non-adeno) | 0.979 | 0.627-1.529 | 0.926 |  |  |  |  |
| Brain metastasis (yes/no) | 1.873 | 1.283-2.736 | 0.001 |  | 2.055 | 1.355-3.115 | 0.001 |
| Liver metastasis (yes/no) | 2.292 | 1.341-3.917 | 0.002 |  | 2.336 | 1.322-4.128 | 0.003 |
| PD-L1 expression (yes/no or unknown) | 0.708 | 0.484-1.034 | 0.074 |  | 0.588 | 0.396-0.874 | 0.009 |
| Driver gene mutation (yes/no or unknown) | 1.117 | 0.751-1.662 | 0.584 |  |  |  |  |
| Corticosteroid use before ICI (yes/no) | 1.552 | 1.069-2.254 | 0.021 |  | 1.494 | 1.005-2.221 | 0.047 |
| Treatment line (1/>1) | 0.627 | 0.426-0.922 | 0.018 |  | 0.688 | 0.463-1.021 | 0.063 |
| ***Overall survival*** |  |  |  |  |  |  |  |
| Sex (Female/male) | 0.772 | 0.447-1.331 | 0.352 |  |  |  |  |
| Age (≥65/<65) | 1.100 | 0.687-1.762 | 0.691 |  |  |  |  |
| Smoking (yes/no) | 0.614 | 0.390-0.966 | 0.035 |  | 0.632 | 0.402-0.996 | 0.048 |
| ECOG PS (>1/0) | 1.887 | 0.866-4.112 | 0.110 |  |  |  |  |
| Histology (adeno/non-adeno) | 0.976 | 0.558-1.705 | 0.931 |  |  |  |  |
| Brain metastasis (yes/no) | 1.006 | 0.623-1.623 | 0.981 |  |  |  |  |
| Liver metastasis (yes/no) | 1.207 | 0.637-2.290 | 0.564 |  |  |  |  |
| PD-L1 expression (yes/no) | 0.501 | 0.312-0.805 | 0.004 |  | 0.513 | 0.320-0.825 | 0.006 |
| Driver gene mutation (yes/no) | 1.042 | 0.628-1.729 | 0.872 |  |  |  |  |
| Corticosteroid use before ICI (yes/no) | 1.218 | 0.767-1.934 | 0.403 |  |  |  |  |
| Treatment line (1/>1) | 0.728 | 0.450-1.176 | 0.194 |  |  |  |  |
| HR: hazard ratio; CI: confidence interval; ECOG PS, Eastern Cooperative Oncology Group performance status; ICI, immune checkpoint inhibitor; No., number. | | | | | | | |

| **Supplemental Table S5. Univariate and multivariate analyses of clinical parameters on progression-free survival and overall survival in patients with BM.** | | | | | | | |
| --- | --- | --- | --- | --- | --- | --- | --- |
| **Factor** | **Univariate analysis** | | |  | **Multivariate analysis** | | |
|  | **HR (log rank)** | **95% CI** | ***P* value** |  | **HR (log rank)** | **95% CI** | ***P* value** |
| ***Progression-free survival*** |  |  |  |  |  |  |  |
| Sex (Female/male) | 2.058 | 1.219-3.475 | 0.007 |  | 1.527 | 0.801-2.907 | 0.199 |
| Age (≥65/<65) | 1.035 | 0.615-1.740 | 0.898 |  |  |  |  |
| Smoking (yes/no) | 0.532 | 0.334-0.848 | 0.008 |  | 0.596 | 0.331-1.074 | 0.085 |
| ECOG PS (>1/0) | 1.408 | 0.609-3.253 | 0.423 |  |  |  |  |
| Histology (adeno/non-adeno) | 0.805 | 0.463-1.400 | 0.442 |  |  |  |  |
| No. of BrMs (<3/>3) | 0.999 | 0.638-1.564 | 0.997 |  |  |  |  |
| Liver metastasis (yes/no) | 1.583 | 0.862-2.909 | 0.139 |  |  |  |  |
| PD-L1 expression (yes/no or unknown) | 0.671 | 0.401-1.122 | 0.128 |  |  |  |  |
| Driver gene mutation (yes/no or unknown) | 1.364 | 0.831-2.240 | 0.220 |  |  |  |  |
| Corticosteroid use before ICI (yes/no) | 1.319 | 0.811-2.147 | 0.265 |  |  |  |  |
| Symptoms at start of ICI (yes/no) | 1.070 | 0.677-1.693 | 0.772 |  |  |  |  |
| Treatment line (1/>1) | 0.794 | 0.471-1.336 | 0.385 |  |  |  |  |
| Treatment (ICI monotherapy/ICI combination) | 1.513 | 0.952-2.404 | 0.080 |  | 1.706 | 1.061-2.745 | 0.028 |
| ***Overall survival*** |  |  |  |  |  |  |  |
| Sex (Female/male) | 1.153 | 0.621-2.139 | 0.652 |  |  |  |  |
| Age (≥65/<65) | 1.187 | 0.632-2.228 | 0.595 |  |  |  |  |
| Smoking (yes/no) | 0.974 | 0.562-1.689 | 0.925 |  |  |  |  |
| ECOG PS (>1/0) | 4.623 | 0.637-33.553 | 0.130 |  |  |  |  |
| Histology (adeno/non-adeno) | 0.831 | 0.439-1.570 | 0.568 |  |  |  |  |
| No. of BrMs (<3/>3) | 0.900 | 0.521-1.554 | 0.705 |  |  |  |  |
| Liver metastasis (yes/no) | 2.229 | 1.146-4.333 | 0.018 |  | 1.234 | 0.599-2.541 | 0.568 |
| PD-L1 expression (yes/no or unknown) | 0.710 | 0.419-1.491 | 0.468 |  |  |  |  |
| Driver gene mutation (yes/no or unknown) | 1.079 | 0.581-2.003 | 0.810 |  |  |  |  |
| Corticosteroid use before ICI (yes/no) | 1.272 | 0.686-2.358 | 0.444 |  |  |  |  |
| Symptoms at start of ICI (yes/no) | 1.038 | 0.596-1.807 | 0.896 |  |  |  |  |
| Treatment line (1/>1) | 0.732 | 0.342-1.563 | 0.420 |  |  |  |  |
| Treatment (ICI monotherapy/ICI combination) | 3.441 | 1.923-6.157 | <0.001 |  | 3.204 | 1.696-6.052 | <0.001 |
| HR: hazard ratio; CI: confidence interval; ECOG PS, Eastern Cooperative Oncology Group performance status; ICI, immune checkpoint inhibitor; No., number; BrMs, brain metastasis. | | | | | | | |
